# Neuron stress-related genes serve as new biomarkers in hypothalamic tissue following high fat diet

**DOI:** 10.3389/fendo.2024.1443880

**Published:** 2024-12-09

**Authors:** Caixia Liang, Hongjian Lu, Xueqin Wang, Jianbin Su, Feng Qi, Yanxing Shang, Yu Li, Dongmei Zhang, Chengwei Duan

**Affiliations:** ^1^ Medical Research Center, Affiliated Hospital 2 of Nantong University, Nantong, China; ^2^ Jiangsu Provincial Medical Key Discipline (Laboratory) Cultivation Unit, Medical Research Center, Nantong First People’s Hospital, Nantong, China; ^3^ Nantong Municipal Medical Key Laboratory of Molecular Immunology, Medical Research Center, Nantong First People’s Hospital, Nantong, China; ^4^ Nantong Municipal Key Laboratory of Metabolic Immunology and Disease Microenvironment, Medical Research Center, Nantong First People’s Hospital, Nantong, China; ^5^ Department of Rehabilitation Medicine, Affiliated Hospital 2 of Nantong University, Nantong, China; ^6^ Department of Endocrinology, Affiliated Hospital 2 of Nantong University, Nantong, China; ^7^ Emergency Intensive Care Unit, Affiliated Hospital 2 of Nantong University and First People’s Hospital of Nantong City, Nantong, China; ^8^ Department of Pathogen Biology, Medical College, Nantong University, Nantong, China

**Keywords:** obesity, neuron stress, hypothalamic inflammation, BMI1, WGCNA

## Abstract

**Objective:**

Energy homeostasis is modulated by the hypothalamic is essential for obesity progression, however, the gene expression profiling remains to be fully understood.

**Methods:**

GEO datasets were downloaded from the GEO website and analyzed by the R packages to obtain the DEGs. And, the WGCNA analysis and PPI networks of co-expressed DEGs were designed using STRING to get key genes. In addition, the single-cell sequencing datasets and GTEx database were utilized to receive the neuron-stress genes from the key genes. Further, high-fat diet (HFD)-induced hypothalamic tissue of mice was used as an animal model to validate the mRNA up-regulation of neuron-stress genes. In addition, the Bmi1 gene was identified as a hub gene through the LASSO model and nomogram analysis. Western blot confirmed the high expression of Bmi1 in hypothalamic tissue of HFD mice and PA-stimulated microglia. Immunofluorescence staining showed that HFD induced the activation of microglia and the expression of Bmi1 in hypothalamic tissue.

**Results:**

We found that six genes (Sacm1l, Junb, Bmi1, Erbb4, Dkc1, and Suv39h1) are neuron stress-related genes and increased in the HFD-induced mice obesity model, Bmi1gene was identified as a key genes that can reflect the pathophysiology of obesity.

**Conclusions:**

Our research depicted a comprehensive activation map of cell abnormality in the obese hypothalamus and Bim1 may be a diagnostic marker in the clinic, which provides a new perspective and basis for investigating the pathogenesis of obesity.

## Introduction

A survey from the World Health Organization in 2016 indicated that 39% of adults worldwide were overweight and 13% were obese. Between 1975 and 2016, the prevalence of obesity nearly tripled worldwide (1). The two main causes of obesity are a high calorie intake and an active-sedentary lifestyle. The battle against obesity has not been effective despite extensive research on the mechanism of obesity by medical professionals and researchers from all around the world. Understanding how our body maintains a healthy weight and what pathological processes interfere with weight control mechanisms can help us treat obesity more successfully. Additionally, it is critical to investigate diagnostic markers that might be used to avoid obesity and its sequelae.

The hypothalamus, which controls the neuroendocrine system as a whole and is known to control energy homeostasis through coordinated actions of neural pathways and neuroendocrine hormones that control energy balance and nutrient homeostasis, regulates food intake and energy expenditure (2–8). Additionally, two neurons that produce neuropeptide Y, agouti-related peptide, and pro-opiomelanocortin (POMC) can combine satiety and hunger signals from peripheral tissue (9). They are the first-line neurons and are responsible for perceiving and responding to metabolic signals affecting the nutritional status in the body (10). Agouti-related peptide (AgRP)-expressing and POMC-expressing neurons reciprocally regulate food intake and can immediately respond to changed glycolipid metabolism. The restricted availability of NPY/AgRP and POMC neurons and narrow technical resources and detecting procedures impedes our comprehension of how these neurons function in processes of obesity.

Elucidating the molecular pathways and cellular functions underlying the development and progression of obesity would contribute to its diagnosis, prevention, and therapy. High-throughput sequencing technology is an effective and strong tool for understanding the pathophysiology of obesity and its comorbidities. In recent years, microarray or sequencing datasets of obesity have been amassed and are available online. The rapid growth of bioinformatics technology, several analysis tools and internet portals have been developed and deployed to uncover disease biomarkers (11–16). Weighted gene co-expression network analysis (WGCNA) can be used to mine linked patterns between genes to discover relevant modules and hub genes for cancer (17). This approach has been frequently utilized to detect biomarkers at the transcriptional level (18, 19). More unexpected is that single-cell transcriptome sequencing has grown rapidly and has been employed in different scientific disciplines in recent years, making it possible to properly describe cell types and associated gene expression profiles in diverse tissues (20–22). Therefore, this new technology innovation has supplied the chance to examine the biomarkers of obesity and its problems.

**Figure 1:**
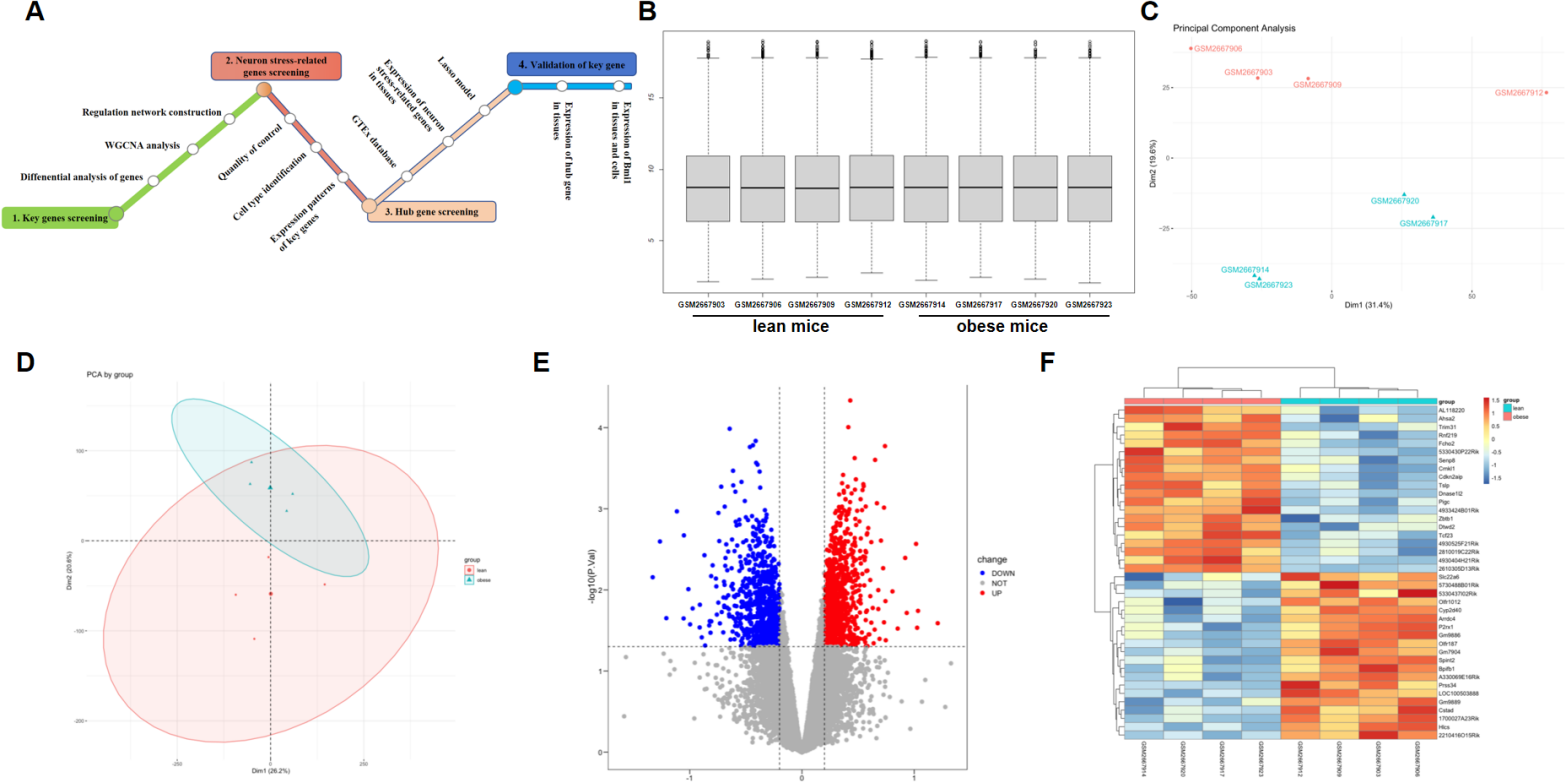
Comparison of DEGs present in obese and lean samples. **(A)** A flowchart showing the steps in this study. Box plots of the gene expression data after normalization. **(B)** The horizontal axis represents the sample symbol, which is divided into a lean and obese group, with four samples in each group, and the vertical axis represents the gene expression values. The black line in the box plot represents the median value of gene expression. **(C, D)** Principal component analysis (PCA) plots showing the expression variability of DEGs across all the samples. The red colour represent lean sample, the green colour represent obese sample. **(E)** The volcano plot for DEGs in the GSE100012 dataset. The X-axes index the -log (P value), and the y-axes index the log fold change. The red dots represent upregulated genes, and the blue dots represent downregulated genes. The gray dots represent genes with no significant difference. FC is the fold change. **(F)** The expression data are represented as a data matrix wherein each row represents a gene and each column represents a sample. The green coded bar above the heatmap represents the lean sample set, and the red coded bar represents the obese sample. The expression level is described in terms of the color ratio of the upper left corner. Hierarchical clustering is shown by the top tree view, indicating the degree of relatedness in gene expression. DEG, differentially expressed genes; FC, fold change.

Based on rich public resources and bioinformatics methodologies, this work discovered six neuron stress-related genes associated with obesity using differential WGCNA and scRNA-seq of GSE100012. We subsequently observed that the mRNA levels of these six neuron stress-related genes were up-regulated utilizing Q-PCR technology in a high-fat diet-induced obesity animal. In addition, LASSO model and nomogram analyses were done to select six neuron stress-related genes and create a diagnostic model for obesity. The Bmi1 gene has a reasonably strong diagnostic performance and provides a new clinical diagnosis biomarker for obesity and associated consequences, which is confirmed by western blot and immunofluorescence staining assays. The workflow of this study is shown in **Figure 1A**.

## Materials and methods

### Gene expression data and processing

GSE100012, the gene expression data for diet-induced changes in mice hypothalamus tissue from lean and obese groups with access to standard chow and high-fat diet (HFD) (23). The dataset was obtained using the Agilent-028005 SurePrint G3 Mouse GE 8x60K Microarray platform and downloaded from the Gene Expression Omnibus (GEO, http://www.ncbi.nlm.nih.gov/geo/). We utilized the R package “limma” to standardize the RNA sequencing data (24). Small variations in gene expression data generally indicate noise, consequently, we used values of coefficient of variation to pick the most variant genes, which were then used to form the network.

### Principal component analysis

Intragroup data repeatability in each group was checked by Pearson’s correlation test. The intragroup data repeatability of the dataset was evaluated using sample clustering analysis. Statistical analysis was performed using the R language, and the findings were presented by the R program “ggplot2”.

### WGCNA network construction and module identification

The WGCNA R software was used to generate the co-expression network (17). First, samples were grouped to check the presence of any evident outliers. Second, the automatic network construction function was employed to generate the co-expression network. The R function choose “Soft Threshold” was used to determine the soft thresholding power β, to which co-expression similarity was elevated to calculate adjacency. Third, hierarchical clustering and the dynamic tree cut function were employed to detect modules. Fourth, gene significance (GS) and module membership (MM) were estimated to tie modules to clinical features. The matching module gene information was retrieved for further study. Finally, we displayed the network of eigengenes.

### Protein–protein interaction network establishment and key gene recognition

PPI network was constructed with Search Tool for the Retrieval of Interacting Genes (STRING; https://string-db.org/), which is one of the largest online databases of known protein‒protein interactions containing the largest number of species (25). The parameter of interactions was set with a confidence score>0.4. The confidence score relates to the strength of data support in terms of the thickness of the line. Thereafter, Cytoscape software (version 3.9.1) was used to generate and evaluate the PPI network (26). Moreover, the Cytoscape plug-in MCODE was utilized to screen important clustering modules in the entire network (27). The Cytoscape plug-in CytoHubba was used to calculate the protein node degree (28, 29). The best three approaches [(Maximal Clique Centrality (MCC), betweenness, and degree] were selected for the analysis. Each method was utilized to demonstrate the respective top 10 genes. A Venn diagram was produced to visualize shared hub genes based on these three methodologies.

### scRNA-seq data analysis

Two public scRNA-seq datasets were retrieved from the database (GSE125065, GSE205667) (30, 31). Before further investigation, we filtered out three interference elements, as follows. First, cells with fewer than 200 identified genes were filtered out, as were cells with a high detection rate (15%) of mitochondrial gene expression. Second, to further exclude probable doublets in our data, cells with more than 6,000 identified genes were additionally filtered away. Third, genes that were discovered in fewer than 10 cells were filtered out. After quality control, the data were normalized and scaled. We reduced the batch impact across distinct individuals by finding anchors between identities and supplied these anchors to the ‘IntegrateData’ method. For data visualization, the dimensionality was further reduced using uniform manifold approximation and projection (UMAP). To cluster single cells by their expression profiles, we employed an unsupervised graph-based clustering technique, Louvain. Cell types were annotated using canonical marker genes. All these analyses were performed by the Seurat(4.0) R package (22). Gene expression in the single-cell datasets was represented by dotplot with percentage of expression and average expression and scattered plot in two µmap dimensions.

### GTEx data analysis

First, the gene expression data matrix in normal hypothalamic samples was retrieved from the GTEX database (Version 8) (https://gtexportal.org/home/), and 157 samples’ expression data were ultimately collected. The TPM value was utilized to demonstrate gene expression and determine the Pearson correlation coefficient between genes. In addition, heatmap was generated to show the expression levels of these genes.

### Animals and treatments

6-8 weeks old male C57BL/6 mice were acquired from the Experimental Animal Center of Nantong University. The animals were kept in a temperature controlled environment with 12 hours light/dark cycle, and free to access water and food. After acclimation for one week, mice were randomly divided into 6 groups (n = 5 per group) and fed either a diet containing 60% kcal fat (HFD, D12492, Research Diets) or a normal chow diet (NCD, D12450J, Research Diets). The experiment was performed for 28 days before all mice were sacrificed and brain tissues were immediately collected for further analyze (14). All animal studies were approved by the Institutional Animal Ethics Committee Nantong University.

### RNA extraction and quantitative Q-PCR

A high-fat diet model was constructed with C57BL/6J mice, and the hypothalamic tissue was stored at -80°C until use (32). RNA samples were extracted from hypothalamic tissues with the Fast Pure Cell/Tissue Total RNA Isolation kit (Vazyme Biotech) using TRIzol reagent (Invitrogen), and they were reverse-transcribed by using 5×PrimeScript RT Master Mix (RR036A,TaKaRa, Japan) according to the manufacturer’s instructions. To determine the relative transcript level, PCR was quantified in realtime using 2×QuantiNova SYBR Green (D7262, Beyotime, China) to analyze gene expression on the Bio-Rad CFX Maestro 1.0 system. The mRNA level was analyzed by using the 2−^ΔΔCT^ method. *Gapdh* was used as a reference gene. The primer pair sequences are following:

*Sacm1l*-F: 5′-GCAGCCTACGAGCATCTGAAG-3′, *Sacm1l*-R: 5′-GGACACTCGGTCAATGATGAGTA-3′;

*Junb*-F: 5′-TCACGACGACTCTTACGCAG-3′, *Junb*-R:5′-CCTTGAGACCCCGATAGGGA-3′;

*Bmi1*-F: 5′-ATCCCCACTTAATGTGTGTCCT-3′, *Bmi1*-R:5′-CTTGCTGGTCTCCAAGTAACG-3′;

*Erbb4*-F: 5′-GTGCTATGGACCCTACGTTAGT-3′, *Erbb4*-R:5′-TCATTGAAGTTCATGCAGGCAA-3′;

*Suv39h1*-F: 5′-GCAGTGTGTGCTGTAAATCTTCT-3′, *Suv39h1*-R:5′-ATACCCACGCCACTTAACCAG-3′;

*Dkc1*-F: 5′-AAAGACCGGAAGCCATTACAAG-3′, *Dkc1*-R:5′-GCCACTGAGAAGTGTCTAATTGA-3′;

*Gapdh*-F: 5′-CAAGGTCATCCATGACAACTTTG-3′, *Gapdh*-R:5′-GTCCACCACCCTGTTGCTGTAG-3′.

### Clinical correlation analysis

Professor. Mara Dierssen had completed various works concerning overweight with high transcriptome sequencing to find the differential expression genes and also shared the original data (23). Thanks to Professor. Mara Dierssen. We employed the original data from the Nestlet shredding test and grooming behavior to explore the significance of hub genes for clinical prediction. The tidy verse, rms, and glmnet programs were used to do lasso regression analysis (33). A nomogram was constructed to predict the prevalence of obesity. Finally, the calibration curve was used to evaluate the accuracy and resolution of the nomogram analysis.

### Cell culture and stimulation

The BV2 microglial cells was cultured in DMEM (11885084, Gibico, USA) supplemented with 10% fetal bovine serum (10091148, Gibico, USA) at 37°C and 5%CO2. Before stimulation, cells were seeded in cell culture dishes overnight, then added with 200μM palmitic acid (PA) (KC004, Kunchuang Biotechnology, China) for the required time, proteins were collected for further analysis.

### Western blot

Hypothalamic tissues and harvested cells were prepared and lysed according to standard protocols as previously described (32). The following antibodies were used: Bmi1 (66161-1-Ig, Proteintech, China), iNOS (18985-1-AP, Proteintech, China), IL-6 (12912, Cell Signaling Technology, USA), α-Tubulin (66031-1-Ig, Proteintech, Chnia). The resulting bands were visualized using the Immobilon Western Chemiluminescent HRP Substrate (PK1001, proteintech, China).

### Immunofluorescence staining

Standard Immunofluorescence procedures were followed. The frozen mouse brain tissue sections were rewarmed and blocked in blocking buffer (10% normal fetal bovine serum, 0.3% Triton X-100) for 2h. Then, the sections were incubated with the primary antibody against Bmi1 and Iba1 (019-19741, FUJIFILM Wako, Japan) followed by 24h at 4°C. Then, the sections were washed with PBS and incubated with the proper Alexa secondary antibodies (A21206, A10037, Thermo Fisher Scientific, USA) 2h at 37°C. Nuclei were stained by Hoechst (33342, Thermo Fisher Scientific, USA) and imaging was performed by fluorescence microscopy (ECLIPSE Ni-E, Nikon, Japan). For each group, three different mouse hypothalamic regions were chosen, and the fluorescence intensity was analyzed using Image J software. The charts were drawn using GraphPad Prism, v8.0 software.

### Statistical analysis

Statistical analyses were performed using GraphPad Prism, v8.0 software. The difference between two groups was analyzed using an unpaired t test, and differences between multiple groups were calculated via analysis of variance (ANOVA) test. Data were expressed as mean ± SEM, and the significance was defined as P < 0.05 (*P < 0.05; **P < 0.01; ***P < 0.001).

## Results

### Research design summary, data normalization and screening of DEGs

Every microarray was adjusted (centered) using quantile data normalization using the bead array package in R Bioconductor. As shown in **Figure 1B**, raw RNA expression data were normalized after preprocessing; median-centered values revealed that the data were normalized, and thus, it was possible to cross-compare between obese and lean samples. The normalized gene expression signals of all expressed genes were utilized to calculate the principal component analysis (PCA) for the identification of the global expression patterns of eight samples. Consequently, the presentation of the top two components demonstrated clear distinction between the lean and obese specimen (**Figures 1C, D**). Significant connection of the biological replicates revealed the quality and trustworthiness of the RNA-seq data and that diet-induced obesity (DIO) might modify the transcriptome profiles.

Batch correction, normalization, and differential analysis of microarray data from GSE100012 were done to test for DEGs in hypothalamus samples. A total of 1814 DEGs, comprising 803 downregulated and 1011 upregulated genes, were discovered from GSE100012 with the screening parameters “adjusted P value<0.05 and FC>0.2”. The results were shown using a volcano plot (**Figure 1E**), which reveals key genes. Furthermore, heatmap calculation confirmed the DEG expression trends revealed in RNA-seq analysis in hypothalamus samples (**Figure 1F**).

### WGCNA and analysis of the most obese-associated module

**Figure 2:**
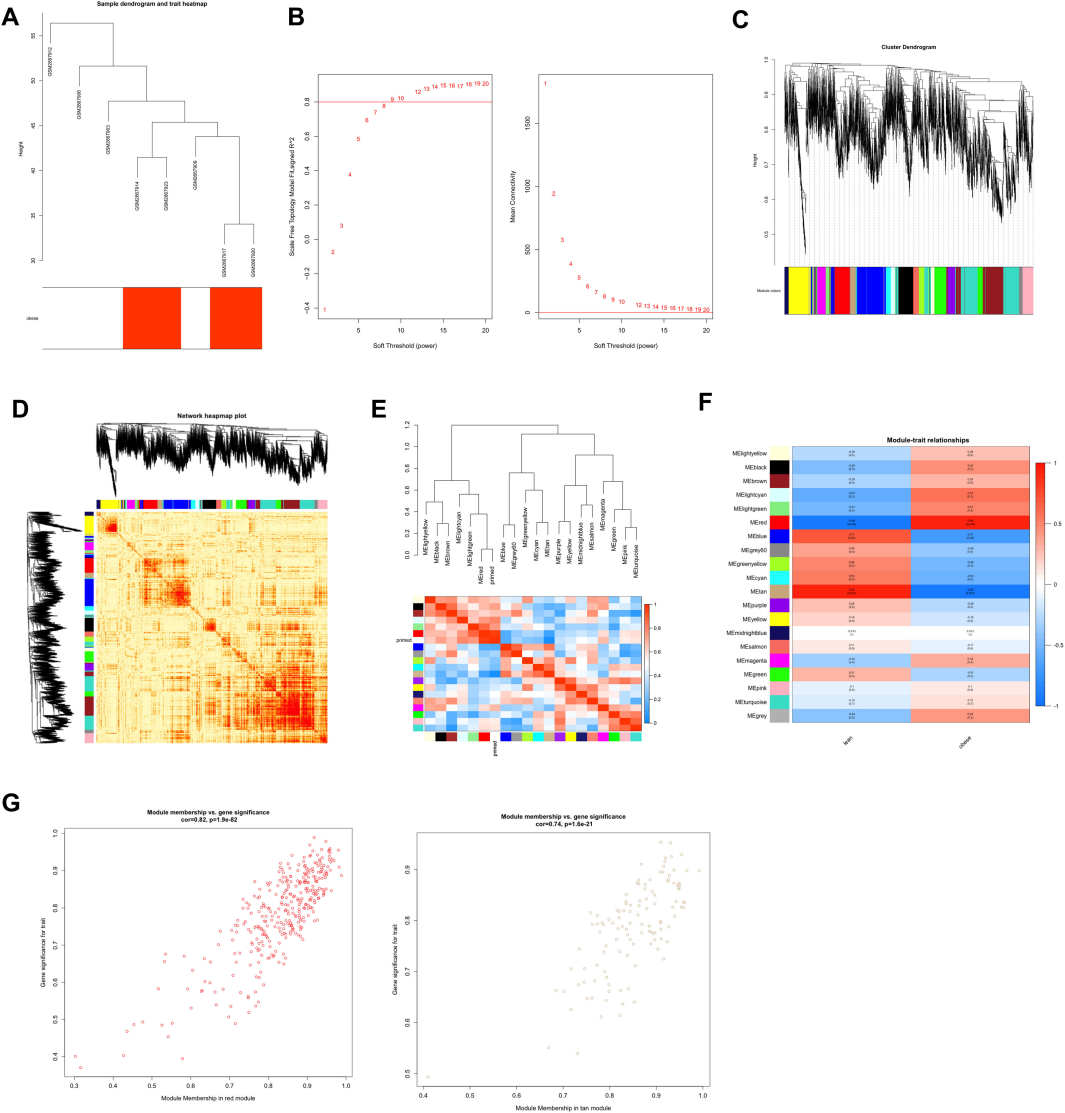
Construction and module analysis of weighted gene coexpression network analysis (WGCNA). **(A)** Sample clustering dendrogram based on Euclidean distance. **(B)** Network topology analysis under various soft-threshold powers. Left: The x-axis represents the soft-threshold power. The y-axis represents the fit index of the scale-free topology model. Right: The x-axis represents the soft-threshold power. The y-axis reflects the average connectivity (degree). **(C)** Clustering dendrogram of genes with different similarities based on topological overlap and the assigned module color. **(D)** The heatmap depicts the topological overlap matrix (TOM) among all modules included in the analysis. The light color represents a low overlap, and the progressively darker red color represents an increasing overlap. **(E)** Eigengene dendrogram and eigengene adjacency plot. **(F)** Module–trait association. Each row corresponds to a module, and each column corresponds to a feature. Each cell contains the corresponding correlation and P value. This table is color-coded according to the relevance of the color legend. **(G)** Scatter plot showing correlations of gene significance for obese vs. module membership in the red and tan modules.

According to the phenotypic characteristics of the high-fat diet, the lean group and the obese group were evaluated by cluster analysis of their gene expression profiles when generating the sample dendrogram. There were no outlier specimens, hence no specimens were deleted (**Figure 2A**). To confirm that the network is scale-free, we used the pick soft threshold function and found that the best soft threshold was automatically established at 9, which makes the evaluation coefficient R2 of the scale-free network run up to 0.8 for the first time (**Figure 2B**).

Next, we created the gene network and identified modules using the one-step network creation function of the WGCNA R package. For cluster splitting, the soft thresholding power was set at 9, the minimum module size was set at 60, and the cutting height was set at 0.25 (which implies a medium sensitivity). Finally, 20 gene co-expression modules were generated (**Figure 2C**). We plotted the links between the indicated modules (**Figure 2D**). The heatmap illustrates the topological overlap matrix (TOM) among all genes included in the investigation. The light color signifies a modest overlap, while the progressively darker red color represents an increasing overlap. The findings of this research suggested that the gene expression was relatively independent amongst modules. We studied the connection of eigengenes. Eigengenes can provide information on the link between paired gene co-expression modules. We clustered the eigengenes and examined their connection. The results showed that 20 modules may be classified into two clusters (**Figure 2E**). The module eigengene (ME) in the red module (r=0.94; p=2e−4) revealed the highest positive correlation, whereas the ME in the tan module (r=-0.92; p=0.001) showed the second highest negative correlation (**Figure 2F**). We connected modules with phenotypic attributes and checked for the most significant associations modules. Both the red (cor=0.82, p=1.2e−92) and tan (cor=0.74, p<1.6e-21) modules revealed strong positive correlations between module member (MM) and gene significance (GS) of the target genes (**Figure 2G**). Therefore, the red module and tan module were designated as essential modules. The modules having a high connection with fat were red and tan.

### GO and KEGG pathways enrichment analyses for the key module

**Figure 3:**
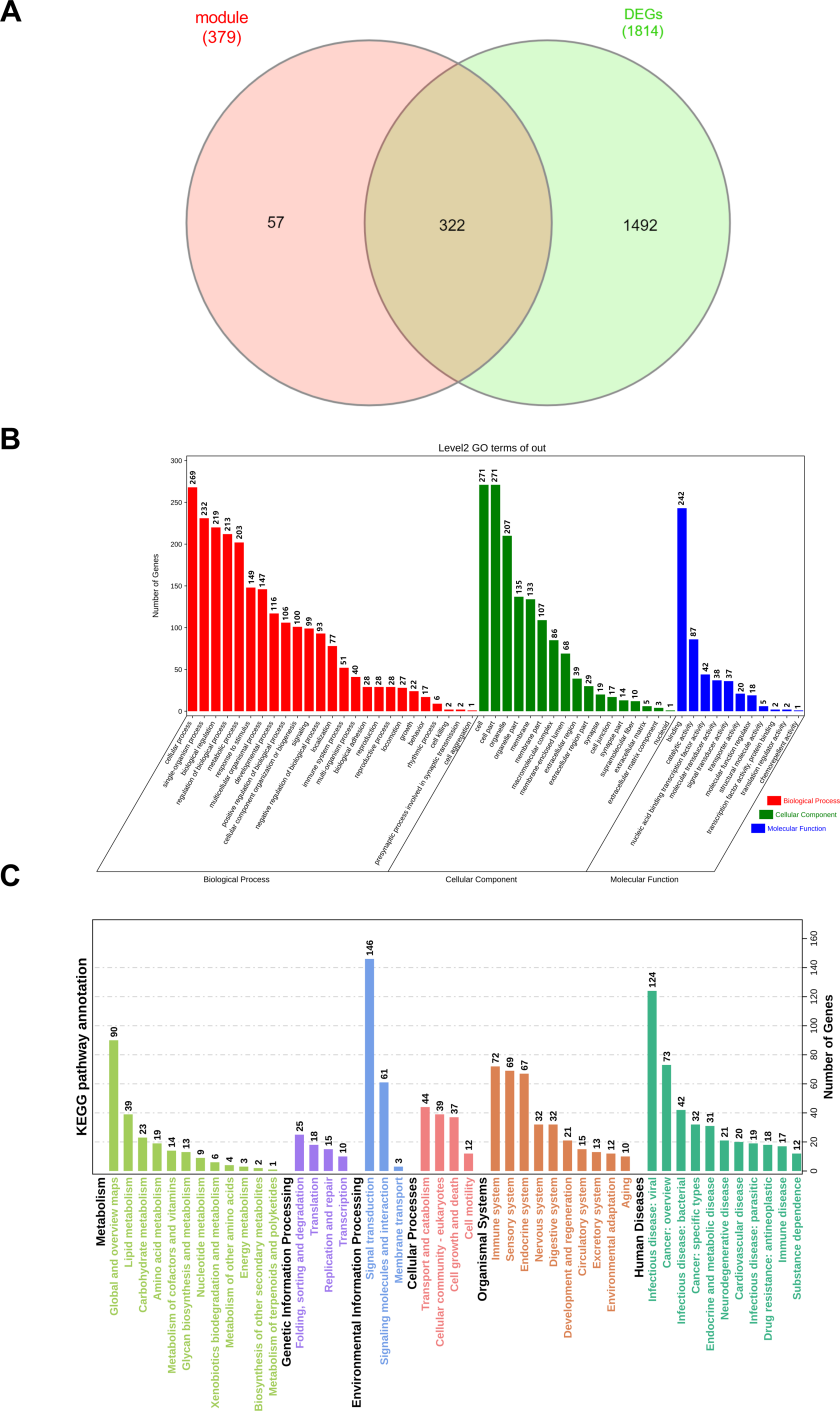
Results of GO enrichment and KEGG analysis. **(A)** The overlapping genes were screening with red and tan modules and DEGs by Venn map. **(B)** The abscissa represents the enriched GO terms, and the ordinate represents the number and ratio of the differentially expressed genes. Different colors represent different GO classes: Molecular function, Biological process, and Cellular component. Abbreviation: GO, gene ontology. **(C)** KEGG bar graph. The related terms were rearranged and classified according to the six classifications of KEGG pathways, and the length of the bar represents the number of gene counts.

Furthermore, 322 overlapping genes were discovered between the red and tan modules and DEGs using a Venn diagram (**Figure 3A**). To investigate the probable biological activities of these genes, GO enrichment studies were done. We did a GO analysis and KEGG analysis of 322 overlapping genes (**Figures 3B, C**). The findings of these analyses showed that, for the biological process, the genes were enriched in response to stimulation, metabolic process, and reproductive process, which are associated to the hypothalamus. Herman et al. has shown that within the hypothalamus, the parvocellular neurons of the paraventricular nucleus (PVN) are a group of densely packed neurons that are highly responsive to external physiological stimuli (34). Lainez et al. discussed the hypothalamic neurons involved in feeding and their interactions with reproductive circuitry. Metabolism influences hypothalamic function and the regulation of GnRH neurons, which are derived from the hypothalamus as the final brain signal that regulates reproduction (35). For the cellular component, overlapping genes were mostly enriched in cell, organelle, and membrane. Regarding molecular function, these genes were strongly associated with binding, catalytic activity, and nucleic acid binding transcription factor activity. The functional analysis (KEGG analysis) of these genes found some regulatory pathways were activated. For example: metabolism pathway (lipid metabolism, carbohydrate metabolism, and amino acid metabolism), genetic information processing (folding, sorting and degradation, translation, replication and repair, transcription), environmental information processing (signal transduction, signaling molecules and interaction, membrane transport), cellular processes (transport and catabolism, cellular community-eukaryotes, cell growth and death, cell motility), organismal systems (immune system, sensory system, and endocrine system), human diseases (infectious disease: viral, cancer: overview, and infectious disease: bacterial).

### PPI network construction and key gene identification

**Figure 4:**
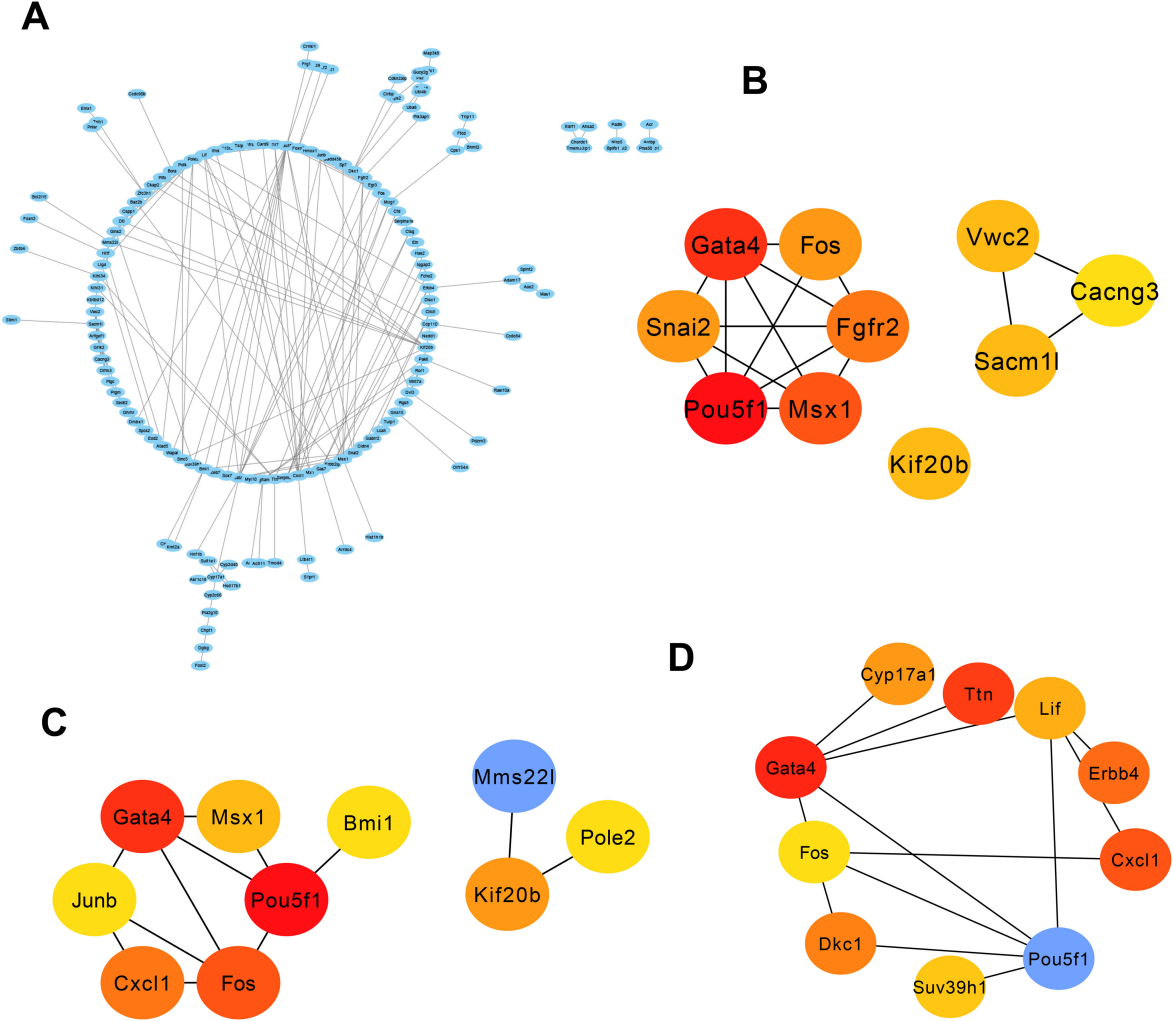
PPI network and three significant modules of the overlapping genes. **(A)** PPI network of overlapping genes created by STRING. The most significant module identified by MCODE. Circles represent genes, and lines represent PPIs. **(B–D)** The top 10 genes were calculated from the PPI network of the DEGs by the degree, MCC, and betweenness. DEG, Deferentially expressed gene; PPI, protein–protein interaction.

We uploaded 322 genes to the STRING online database to obtain the PPI network. The generated PPI network comprised a total of 266 nodes and 178 edges. **Figure 4A** provides the depiction of the network built using Cytoscape software (version: 3.9.1). Subsequently, neuron stress-associated genes were found by using the Cytohubba component of Cytoscape program. Next, we picked three algorithms to screen the neuron stress-associated genes in the PPI network and identified 30 key genes. As shown in **Figures 4B–D**, the top 10 genes were identified by degree (*Pou5f1, Gata4, Fos, Cxcl1, Kif20b, Msx1, Junb, Pole2, Mms22l, Bmi1*), MCC (*Pou5f1, Gata4, Msx1, Fgfr2, Fos, Snai2, Sacm1l, Kif20b, Vwc2, Cacng*3), and betweenness (*Pou5f1, Gata4, Ttn, Cxcl1, Erbb4, Dkc1, Cyp17a1, Lif, Suv39h1, Fos*).

### ScRNA-Seq data revealed high cell heterogeneity in hypothalamic tissue and neuronal stress-related gene identification

**Figure 5:**
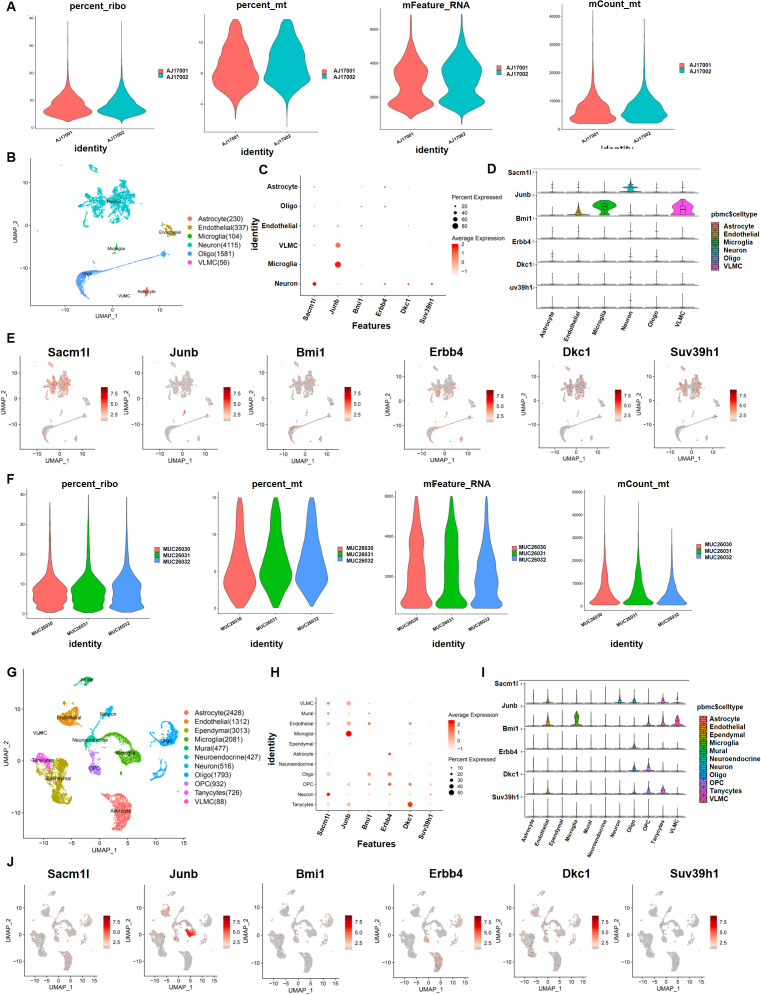
Preprocessing of the single-cell sequencing data and cell cluster identification with GSE125065 and GSE205667. First, with GSE125065: **(A, B, F, G)** Gene filtering and PCA clustering of the gene expression matrix. **(C, H)** Dot plot showing the expression of overlapping genes in each main cell type. The darker color indicates higher expression, and the larger size represents a higher percentage of expression. **(D, E, I, J)** Expression pattern of six neuron stress-related genes at the single-cell level, shown in violin and UMAP plots.

Because of the variability of hypothalamic tissue, we determined whether the 30 neuron stress-associated genes play a significant role in neuron cells in the DIO model. We utilized scRNA-sequencing data from hypothalamic tissues that were treated with NCD and HFD to study which cells these 30 neuron stress-related genes are mostly found in to operate. We initially conducted quality control of the gene expression matrix using the GSE125065 dataset (**Figure 5A**). Then, normalization of scRNA-seq data was conducted, and 20 main components (p<0.05) were screened for subsequent analysis (**Figure 5B**). Reduced dimension process analysis was done by employing a discriminative dimensionality reduction tree (**Figure 5C**). Unsupervised analysis was subsequently undertaken for cell clustering using the t-distributed stochastic neighbor embedding (t-SNE) approach (**Figure 5D**). The results demonstrated substantial cell heterogeneity, in which hypothalamic cells were segregated into six major different clusters, including astrocytes, microglia, neurons, oligodendrocytes, vascular leptomeningeal cells (VLMCs), and endothelial cells, which were detected by single R and cell markers. Next, we investigated the expression pattern of these 30 genes in these cell clusters. As expected, six critical genes of these 30 key genes were substantially expressed in neurons, including Sacm1l, Junb, Bmi1, Erbb4, Dkc1 and Suv39h1 (**Figure 5E**). In addition, we utilized another GSE205667 dataset to verify the enrichment of these 30 genes in different cell lines in the arcuate nucleus of the hypothalamus (ARC) (31). Similar to the analysis method and R package, the data are displayed in **Figures 5F–J**. The results showed that these six genes were enriched in small amounts in neuron cells in ARC at different time points in adaptation to a high-calorie diet. Erbb4 was enriched in astrocytes and Junb was mainly enriched in microglia. Taken together, the scRNA-Seq data showed that these six genes (Sacm1l, Junb, Bmi1, Erbb4, Dkc1, and Suv39h1) were basically expressed in the hypothalamic region of mice, but were not mainly enriched in neuronal cells.

### Neuron stress-associated gene expression in human hypothalamus tissue

**Figure 6:**
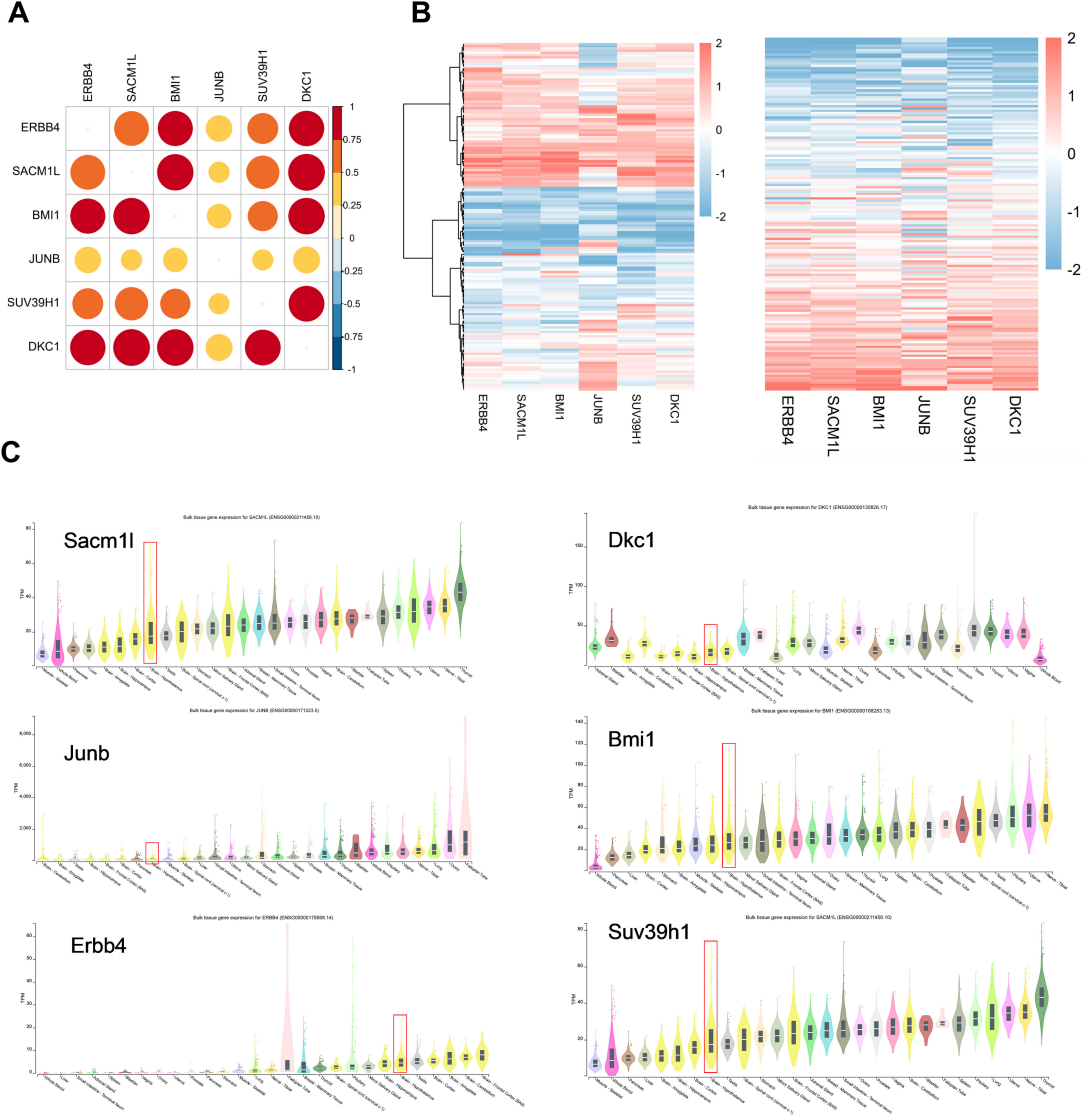
Neuron stress-related gene expression in the human hypothalamus. **(A)** A correlation heatmap of neuron stress-related gene expression in 121 human hypothalamic tissue samples. **(B)** Heatmap of coexpression correlations between neuron stress-related genes. A darker red color in the upper right part indicates a stronger correlation. A heatmap of neuron stress-related gene expression levels is shown in the right panel. **(C)** Expression of Neuron stress-related genes in different tissues, as the violin plots show.

To evaluate whether these six neuron stress-associated genes listed above are relevant in humans, we employed RNA-seq data from 121 human hypothalamus tissues from the Genotype-Tissue Expression (GTEx) project to assess the expression levels of six neuron stress-associated genes (36). The correlation heatmap and expression heatmap utilizing unbiased hierarchical clustering revealed that six neuron stress-associated genes were notably highly expressed (**Figures 6A, B**). Furthermore, we further evaluated the expression of the six neuron stress-associated genes in different tissues of the GTEx database and found that they were all expressed in human hypothalamus tissue (**Figure 6C**).

### Corroboration of hub genes using Q-PCR in hypothalamic tissues in the DIO model

**Figure 7:**
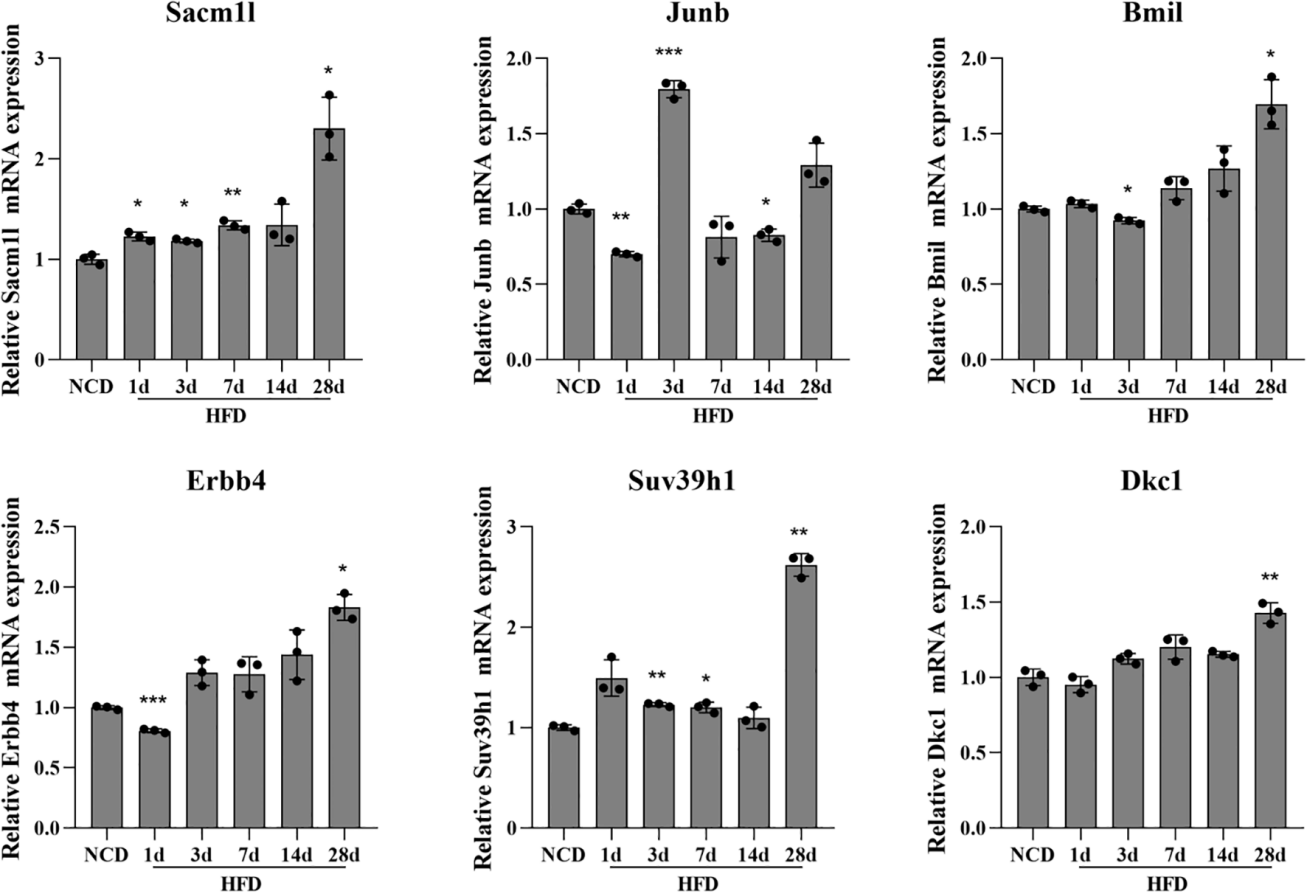
Differential expression of neuronal stress-related genes in the HFD-induced hypothalamic tissue model of obesity. Relative mRNA expression of neuron stress-related genes measured by Q-PCR in the HFD-induced hypothalamic tissue model of obesity. Results are presented as mean ± SEM (n=3). The differences between multiple groups were evaluated using one-way analysis of variance (ANOVA). Compared with NCD group: *P<0.05, **P<0.01, ***P<0.001. NCD, normal chow diet group; HFD, high-fat diet group.

Q-PCR was done using the total RNA isolated from the hypothalamus tissue in the DIO model to confirm the expression levels of neuron stress-associated genes. According to the PPI network analysis and scRNA-Seq data validation, the six key genes tested were Sacm1l, Junb, Bmi1, Erbb4, Dkc1 and Suv39h1. The expression levels of the six neuron stress-associated genes were raised as part of the pathophysiological process of obesity. The findings of the Q-PCR experiment demonstrated a considerable increase in the expression of the six neuron stress-associated genes (**Figure 7**).

### Diagnostic value of Bmi1

**Figure 8:**
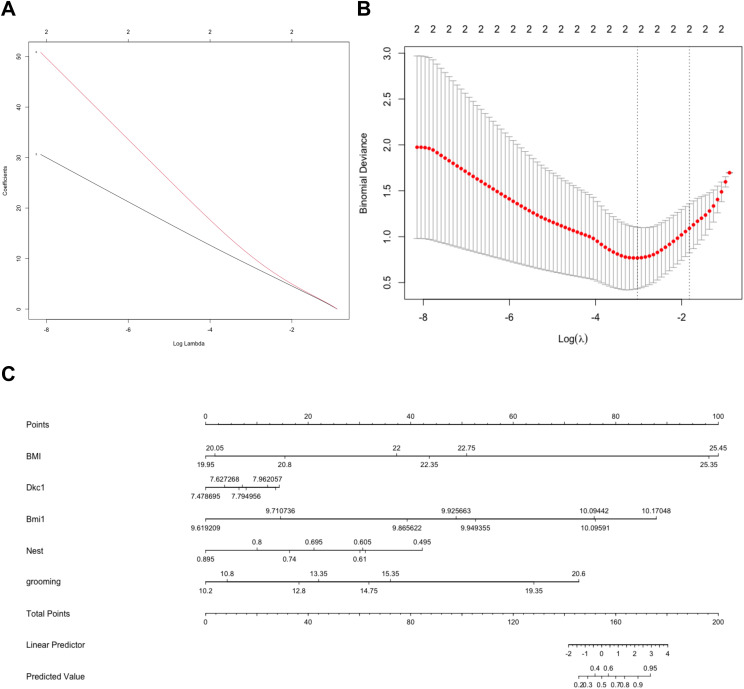
Investigation of the accuracy of the hub genes for distinguishing Obese samples from lean samples. Construction of the LASSO model based on 6 candidate biomarkers **(A)**. The image shows the log (lambda) value of the 2 hub genes, and **(B)** the image shows the distribution of the log (lambda) value in the LASSO model. **(C)** The nomogram was used to predict the occurrence of obesity.

To investigate the accuracy of the genes for discriminating the obese samples from lean samples, we first created a LASSO model utilizing the six candidate genes (**Figures 8A, B**). The nomogram was employed for predicting the occurrence of obesity (**Figure 8C**). These results revealed that Bmi1, rather than the Dkc1 gene, possessed outstanding accuracy in identifying obese from lean samples.

### HFD induces microglial activation and Bmi1 expression

**Figure 9:**
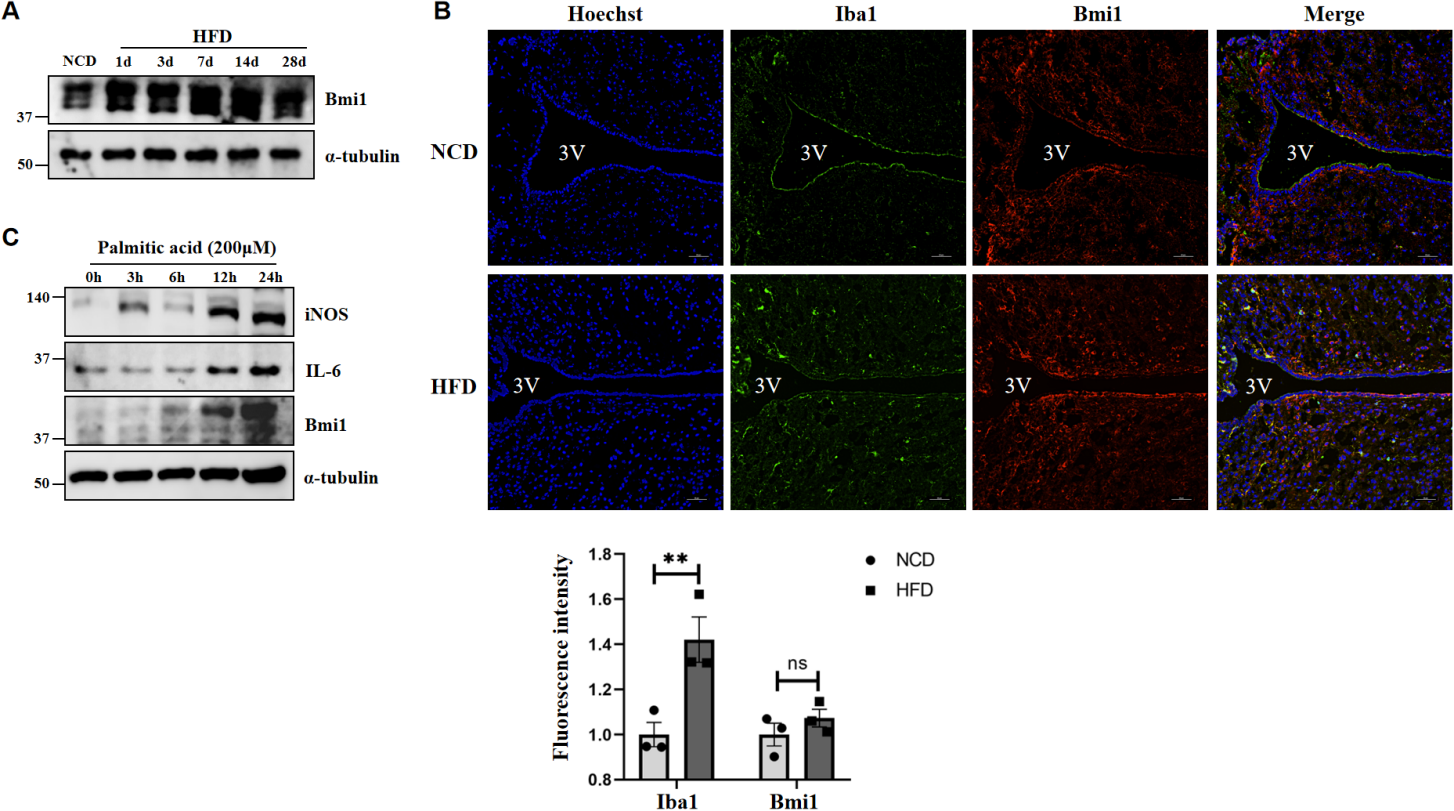
A high-fat diet induces hypothalamic microglia activation and Bmi1 expression. **(A)** The protein expression of Bmi1 was analyzed by western blot in the HFD-induced hypothalamic tissue model of obesity. **(B)** Immunofluorescence staining was used to observe the expression of Iba1 and Bmi1 in the hypothalamus of mice. The fluorescence intensity was normalized to that of each group fed with NCD group set as 1. The data were obtained from three independent experiments. Values represent the mean ± SEM. **p <.01; ns, no significance by two-way analysis of variance (ANOVA). Scale bar: 50 μm. NCD, normal chow diet group; HFD, high-fat diet group; 3V, the third ventricle. **(C)** Western blot was used to analyzed the expression of iNOS, IL-6, and Bmi1 proteins after PA administration in BV2 microglial cells.

Furthermore, we detected the expression of Bmi1 in hypothalamic tissue of the DIO model by western blot, confirming that the expression of Bmi1 was significantly increased following HFD administration (**Figure 9A**). From the results of **Figures 5D, I**, we found that in addition to neuron cells, Bmi1 is also highly enriched in hypothalamic microglia. Immunofluorescence staining showed that the positive number of microglia (Iba1, microglia activation marker) in the hypothalamus was significantly increased at 3 days in the HFD group, and there was colocalization with Bmi1 (**Figure 9B**). In addition, we stimulated BV2 microglial cells with palmitic acid (PA, 200μM) *in vitro*, and western blot further confirmed the elevated expression of iNOS, IL-6, and Bmi1 proteins after PA administration (**Figure 9C**). The above results indicated that HFD induces microglial activation and Bmi1 expression in hypothalamic tissue.

## Discussion/conclusion

In the current study, we combined WGCNA, integrated bioinformatics, PPI network, scRNA-seq and the GTEx database to identify *Sacm1l*, *Junb*, *Bmi1*, *Erbb4*, *Dkc1* and *Suv39h1* as neuron stress-related genes from eight samples of the GSE100012 dataset. Further LASSO model and nomogram analysis showed the Bmil gene to be the hub gene by screening six neuron stress-related genes. In addition, Q-PCR and western blot revealed that the mRNA and protein level of Bmil were raised in the DIO model. These discoveries will throw new light on possible diagnostic tools to manage obesity.

In previous report, research by professor Mara Dierssen’s team has shown that obesogenic diets can affect the transcriptome changes in hypothalamus, frontal cortex, and striatum, which are involved in feeding and energy balance (23). We utilized the team’s dataset for our research, and we are grateful for their great contribution. However, compared with the work of this team, there are some differences. First, professor Mara Dierssen’s team utilized the female mice (five weeks) and chocolate mixture which provides 20595 kJ/kg with 52% of its energy from carbohydrate, 17% from protein, and 24% from fat. But, our team utilized the male mice (6-8 weeks) and HFD (60% kcal fat). As we all known, long term intake of foods with different fat contents will definitely affect the metabolism of the body. Also, in some studies, female mice gained significantly less weight than male mice when given the same HFD, indicating a resistance to diet-induced obesity. Research has also shown sex differences in gut microbiome composition between males and females, indicated to be in part a result of sex hormones (37). Compared the work from professor Mara Dierssen’s team, our research from different angels to explore the transcriptome changes of hypothalamus and identified the hub genes with diet induced obesity model. In addition, the work of professor Mara Dierssen’s team construct two groups of genes: some transcript were strongly deregulated in term of fold changes, while others were only subtly deregulated but were especially correlating with measurements associated with body weight and compulsivity. These results told us that even if some genes show no or minimal changes in expression, they can still affect feeding behavior in the hypothalamic area following overeating. Our research not only identified key genes Bmil1, but also verified the function of Bmil1 through animal and cell models which can regulate the inflammatory activation of hypothalamic microglia. Compared the work of professor Mara Dierssen’s team, our data exhibit an improvement.

Obesity develops as a result of equilibrium disruption between food intake and energy consumption, and this balance is principally governed by two well-defined neuronal populations within the ARC that have significant effects on energy homeostasis, namely, POMC and AgRP/NPY. Rodents on a high-fat diet exhibit hypothalamic inflammation, gliosis, and neuronal stress. These changes happened in the arcuate nucleus, mediobasal hypothalamus, and lateral hypothalamus, thereby demonstrating that these hypothalamic structures are targets of brain damage (38, 39). Metabolic derangement in peripheral organs and obesity-related inflammatory alterations cause hypothalamic neuron stress that perturbs energy balance and affects hormone release, which eventually promotes the establishment of an obese phenotype. Therefore, it is very relevant to examine neuronal stress indicators following DIO. Based on bioinformatics technology, we observed that the Bmil gene is closely associated with neuronal stress and has an increase in expression at the mRNA level. *Bmi1* (also named as B-lymphoma Moloney murine leukemia virus insertion region-1), as a member of the polycomb-group (PcG) family of proteins, is increased in various human cancer tissues and can regulate cell malignant transformation, cell proliferation and apoptosis and cell cycle (40). *Bmi1^−/−^* mice not only have axial skeleton deformities, lower lifespan, cortical neuron apoptosis, and oxidative damage buildup, but Bmi1^−/−^neurons are also hypersensitive to mitochondrial toxins and demonstrate higher ROS concentrations (41–43). Notably, *Bmi1* could modulate the neuron oxidative metabolism via the p53 signaling pathway (44). Increasing *Bmi1* expression in cortical neurons demonstrated neuroprotective activity through resisting inhibition of the mitochondrial respiratory chain and finally led in activation of antioxidant defenses and down-regulation of ROS levels (45). To yet, no study has directly established the involvement of *Bmil* in obesity and another large range of comorbidities. Further work is necessary to clarify the underlying biological mechanisms.

Obesity has been recognized to cause systemic chronic low-grade inflammation condition in the central and peripheral system that contributes to the development of multiple comorbidities, such as diabetes, dyslipidemia, cardiovascular diseases, and neurodegenerative disorders (46–48). This low-grade inflammation can produce neuronal stress and alter the hypothalamic circuit of energy balance, which leads to a vicious cycle of obesity in the hypothalamic tissue. Hypothalamus inflammation was initially discovered in 2005 because inflammatory signaling pathways were activated and the mRNA levels of proinflammatory factors also elevated in the hypothalamus of rats fed a high-fat diet (HFD) for 16 weeks (49).

More recently, hypothalamic inflammation has been found to occur very early in response to HFD and is the main trigger to promote the development and progression of obesity and its sequelae and has emerged not only as an important driver of impaired energy balance but also as a contributor to obesity-associated insulin resistance via altering neurocircuit functions. Hypothalamic inflammation plays an important role in the onset and maintenance of the obese phenotype (50). It has been reported that hypothalamic inflammation presents a “double peak”, which is manifested in three stages: “inflammation initiation, inflammation resolution, and chronic inflammation”. Hypothalamic inflammation increases rapidly in the early days (1-3 days) of high-glucose and high-fat feeding, briefly resolves at 7-14 days, and then rises again and persists after 28 days. The transient spontaneous resolution of hypothalamic inflammation suggests a physiological mechanism for maintaining hypothalamic homeostasis, and the disruption of this mechanism may be the key to the persistence of inflammation and the progression of obesity (38, 51). Therefore, the expression of neuron stress-related genes (Sacm1l, Junb, Bmi1, Erbb4, Dkc1, and Suv39h1) fluctuates at different time points. Compared with the NCD group, the expressions of Sacm1l, Bmi1, Erbb4, Dkc1 and Suv39h1 showed a fluctuating upward trend, with the most significant change on day 28. While the expression of Junb increased most significantly on day 3, and then showed a downward trend (**Figure 7**). These results suggest that these genes may be involved in the regulation of hypothalamic inflammation and homeostasis. The interrelations between inflammation, hypothalamus, and obesity have been extensively reviewed (52, 53). We also performed immune infiltration analysis to explore the relationship between the immune landscape and obese samples from GSE100012 (data not shown). The CIBERSORT method was used with a deconvolution algorithm, which was applied to estimate the relative proportion of 25 types of immune cells among the two groups (54). These data cannot properly reflect the expression status and fraction of immune cells, and further analysis needs to be performed with other datasets. In addition, the GO and KEGG pathway analyses revealed that the biological functions of the red and tan modules were strongly enriched for response to stimulate, locomotion, rhythmic process, reproductive process, lipid metabolism, endocrine system, and sensory system, which are modulated by the hypothalamus. Enrichment function analysis indicated a contributory role of the immune response in the development of obesity. Taken together, how hypothalamic inflammation impacts neuronal stress and increases the occurrence of obesity remains unclear and is also an exploring direction in our lab.

This study had significant drawbacks. Through examining the GEO database, there are merely handful datasets about hypothalamus tissue with a HFD. A bigger sample size will be needed in future investigations, and we also want to utilize mouse hypothalamus tissue after a high-fat diet to do transcriptome sequencing. Moreover, functional studies of the six neuron stress-related genes discovered here are essential.

In summary, we created a WCGNA, PPI network, and gene regulatory network to detect and validate target genes as biomarkers for obesity. GO and pathway enrichment studies suggested that the biological functions of the overlapping genes between the red and tan modules and DEGs were geared toward hypothalamic homeostasis. Moreover, our data indicated considerably elevated expression of *Sacm1l*, *Junb*, *Bmi1*, *Erbb4*, *Dkc1*, and *Suv39h1*. Furthermore, the scRNA-seq and GTEx databases indicated that the six genes were largely localized in neuron-type cells. Finally, LASSO model and nomogram analysis suggested that Bmi1 was a significant hub gene, and Q-PCR and western blot confirmed that Bmil was increased in the DIO model. If there is a need to understand their activity in obesity, as well as their molecular mechanisms of action in obesity and related metabolic diseases, further experiments are needed.

## Data Availability

The original contributions presented in the study are included in the article/supplementary material, Further inquiries can be directed to the corresponding author/s.
